# Cartilage oligomeric matrix protein deficiency promotes early onset and the chronic development of collagen-induced arthritis

**DOI:** 10.1186/ar2551

**Published:** 2008-11-14

**Authors:** Hui Geng, Stefan Carlsen, Kutty Selva Nandakumar, Rikard Holmdahl, Anders Aspberg, Åke Oldberg, Ragnar Mattsson

**Affiliations:** 1 Department of Experimental Medical Sciences, BMC, Lund University, Sölvegatan 19, SE-22184 Lund, Sweden; 2 Current address: Department of Biochemistry and Molecular Biology, College of Life Science, Huazhong Normal University, No. 100 Luoyuroad, Wuhan 430079, PR China; 3 Department of Biology, University of Copenhagen, Biocenter, Ole Maaløes Vej 5, DK-2200 Copenhagen, Denmark

## Abstract

**Introduction:**

Cartilage oligomeric matrix protein (COMP) is a homopentameric protein in cartilage. The development of arthritis, like collagen-induced arthritis (CIA), involves cartilage as a target tissue. We have investigated the development of CIA in COMP-deficient mice.

**Methods:**

COMP-deficient mice in the 129/Sv background were backcrossed for 10 generations against B10.Q mice, which are susceptible to chronic CIA. COMP-deficient and wild-type mice were tested for onset, incidence, and severity of arthritis in both the collagen and collagen antibody-induced arthritis models. Serum anti-collagen II and anti-COMP antibodies as well as serum COMP levels in arthritic and wild-type mice were measured by enzyme-linked immunosorbent assay.

**Results:**

COMP-deficient mice showed a significant early onset and increase in the severity of CIA in the chronic phase, whereas collagen II-antibody titers were similar in COMP-deficient and wild-type controls. COMP antibodies were not found in wild-type mice. Finally, COMP-deficient and wild-type mice responded similarly to collagen antibody-induced arthritis, indicating no difference in how collagen II antibodies interact with COMP-deficient cartilage during the initial stages of arthritis.

**Conclusions:**

COMP deficiency enhances the early onset and development of chronic arthritis but does not affect collagen II autoimmunity. These findings accentuate the importance of COMP in cartilage stability.

## Introduction

Rheumatoid arthritis (RA) is a human autoimmune disease that affects the synovial membranes of the peripheral joints. RA characteristically involves the infiltration of leukocytes into the synovium, which undergo inflammation and swelling [1]. RA in humans is a heterogenous disease, and the environmental and genetic factors involved in its manifestation and perpetuation may vary from individual to individual. Although there is an HLA association, the classification of RA is based primarily on diagnostic criteria [2], of which the presence of joint swelling in the hands, the duration and symmetry of the joint swelling, and abnormal levels of rheumatoid factors are some examples. More recently, it has been shown that the serum levels of cartilage oligomeric matrix protein (COMP) are elevated in a high proportion of patients suffering from RA [3-5], which not only is of diagnostic interest but also may indicate that this cartilage protein is involved in the disease process.

In 1977, Trentham and colleagues [6] developed the collagen-induced arthritis (CIA) model in rats as a model for the study of RA. Since then, several modified CIA models have been developed in mice, and CIA is still one of the most common animal models of RA. The CIA model shares some important features with RA, namely major histocompatibility complex (MHC) association and the fact that peripheral joints primarily are affected and that the disease can be divided into an acute and a chronic stage. In the CIA model, an immunization against one specific cartilage protein, collagen II (CII), starts an autoimmune reaction leading to arthritis. In the chronic stage of the disease, when the erosion of the cartilage is taking place, it is possible that immune reactions to other cartilage proteins are initiated and contribute to the disease course. In fact, the immunization with other cartilage proteins in some cases will cause a disease similar in type to CIA, which has been shown to be immunized with COMP [7].

COMP is a 524-kDa homopentameric extracellular matrix glycoprotein and a member of the thrombospondin (TSP) family [8]. To date, five members of the TSP family have been identified. Among them, TSP-1 and TSP-2 contain three identical subunits [9-12], whereas TSP-3, TSP-4, and COMP (also called TSP-5) contain five identical subunits [8,13,14]. COMP is present in cartilage, tendon, vitreous of the eye, and vascular and smooth muscle cells [15,16]. In adult articular cartilage, COMP is most abundant in the inter-territorial matrix [17]. As previously mentioned, COMP has recently been found to be a useful biomarker for pathological conditions since the detection of COMP fragment levels in synovial fluid or serum can be used to assess the presence and progression of arthritis [3-5]. The importance of COMP for cartilage structure and function is underscored further by the findings that COMP mutations cause human skeletal dysplasia, pseudoachondroplasia (PSACH), and multiple epiphyseal dysplasia (MED) [18,19]. The clinical features of PSACH and MED are mild to severe short limbs, joint laxity, and early osteoarthritis. Although the clinical features are similar, PSACH is normally more severe than MED [20-22].

We have previously generated COMP-deficient mice to study the role of COMP in cartilage tissues [23]. Surprisingly, the total absence of COMP did not result in an obvious phenotype, and the COMP-deficient mice did not show any abnormalities in their cartilage and skeletal tissues [23]. The function of COMP in cartilage remains unknown. Interestingly, mice deficient in the cartilage matrix protein collagen type IX, which (like COMP-deficient mice) appear phenotypically normal, showed cartilage properties significantly different from those of the wild-type mice when investigated in the CIA and CII antibody-induced arthritis (CAIA) models. Apparently, the microstructure of the cartilage of collagen IX-deficient mice had changed in a way that anti-CII antibodies could more easily reach immunogenic CII epitopes, which in turn caused a more severe arthritis in the acute stage of the disease [24]. Thus, the absence of one cartilage protein can affect how effector molecules of the immune system reach and bind other cartilage proteins.

To test whether COMP deficiency, like collagen IX deficiency, influences the antigenic/immunogenic properties of the cartilage, we decided to study COMP-deficient mice in the CIA and CAIA models. In this paper, we present results indicating that COMP deficiency makes arthritic mice develop an early onset and more severe disease during the chronic phase. We also present data showing that the exacerbation of the disease in arthritic COMP-deficient mice is independent of how pathogenic antibodies penetrate the cartilage in the acute stage of the disease, which is contrary to the case in collagen IX-deficient mice [24]. Finally, a role for COMP in the cartilage repair mechanism is discussed as a possible explanation for the exacerbation of the chronic stage of the disease in COMP-deficient mice.

## Materials and methods

### Animals

The generation of COMP-deficient 129/Sv mice has been described previously [23]. COMP-deficient 129/Sv mice were backcrossed for 10 generations to B10.Q mice (originally obtained from The Jackson Laboratory, Bar Harbor, ME, USA), which are susceptible to CIA. The mice were kept in a climate-controlled environment with 12-hour light-dark cycles, housed in polystyrene cages containing wood shavings, and provided with standard rodent chow and water *ad libitum *in the animal house of the Department of Pathology, Lund University (Lund, Sweden). All experiments described here were performed on age-matched mice between 8 and 10 weeks of age. The Lund-Malmö laboratory animal ethics committee approved the animal experiments described in this article.

### Induction and evaluation of collagen II-induced arthritis

The mice were injected subcutaneously at the base of the tail with 100 μg of rat CII emulsified in 0.1 M acetic acid combined with an equal amount of complete Freund's adjuvant (Difco Laboratories, now part of Becton Dickinson and Company, Franklin Lakes, NJ, USA). CII was purified from the Swarm rat chondrosarcoma as previously described [25]. At day 30 after primary CII immunization, a booster injection of 50 μg of rat CII in incomplete Freund's adjuvant was given at the same location. Arthritis development was monitored in all four limbs by means of a macroscopic scoring system [26]. Briefly, one point was given for each swollen or red toe, one point for each swollen joint (metatarsal phalangeal joints, metacarpal phalangeal joints, proximal interphalangeal joints, and distal interphalangeal joints), and five points for a swollen ankle (maximum score per limb was 15 and maximum score per mouse was 60). The mice typically were examined three times per week up to 5 months after immunization.

### Induction and evaluation of collagen II antibody-induced arthritis

To induce CAIA, the mice were injected with a mixture of equal concentrations of sterile filtered CIIC1 (IgG2a), M2139 (IgG2b), CIIC2 (IgG2b), and UL1 (IgG2b) monoclonal antibodies against different CII epitopes (C1, J1, D3, and U1) [27]. Mice were injected intravenously with 0.33 mL of antibody mixture as a single dose on day 0. Subsequently, on day 8, lipopolysaccharide (LPS) from *Escherichia coli *055:B5 (25 μg/mouse) was injected intraperitoneally to enhance the incidence and severity of arthritis. The mice were monitored daily for arthritis development after antibody injection (both before and after LPS injection), using the same macroscopic scoring system as described above for CIA.

Production of recombinant mouse cartilage oligomeric matrix proteinA mouse COMP cDNA clone was kindly provided by Liu Chan Ju (Department of Orthopaedic Surgery, New York University Hospital for Joint Diseases, New York, NY, USA). A cDNA fragment corresponding to nucleotides 72 to 2,282 in the mouse COMP reference sequence [GenBank NM016685] and comprising the entire COMP open reading frame, except the signal peptide, was amplified by polymerase chain reaction (PCR), using primers mCOMP-TNT-F (5'-CAGGGCCAGATCCCGCTG-3') and mCOMP-TCG-R (5'-CGTGCTAGCCTAAACTCTCTGCAGCC-3'), introducing a downstream *Nhe *I restriction site. The PCR product was subcloned into plasmid pCR-SCRIPT and sequenced. This revealed a mutation (C493T, Thr160Ile) compared with the reference sequence. This may represent a naturally occurring allele, but since the mutated residue is conserved in human, chimp, bovine, equine, and rat COMP, the cDNA sequence was corrected by site-directed mutagenesis using the QuikChange kit (Stratagene, La Jolla, CA, USA) and primers FwdMUT (5'-CCCCCTGGGTTCAGCGGGCCCACCCACGAGGGCGTGGGACTGACC-3') and RevMUT (5'-GGTCAGTCCCACGCCCTCGTGGGTGGGCCCGCTGAACCCAGGGGG-3'). The corrected COMP cDNA fragment was isolated by digestion with *Bgl *I and *Not *I restriction enzymes and ligated into the corresponding sites in the expression vector pCEP4-BM40-hisEK. The resulting mouse COMP expression plasmid was transfected into 293-c18 cells (ATCC CRL-10852) and selected with hygromycin. Afterwards, conditioned medium was collected and the his-tagged recombinant mouse COMP was purified through Ni^2+^-metal chelating and MonoQ ion exchange chromatography. Protein content was determined by measuring absorbance at 280 nm, using a calculated extinction coefficient of 71,390/M per cm.

### Determination of serum levels of antibodies against cartilage oligomeric matrix protein and collagen II

Antibody levels against COMP in serum were analyzed by enzyme-linked immunosorbent assay (ELISA) using recombinant mouse COMP. Recombinant COMP (50 μL/well; 5 μg/mL in phosphate-buffered saline (PBS), pH 7.4) was used for coating overnight at 4°C, and plates were pre-blocked with 1% bovine serum albumin in PBS to avoid background disturbance. All washings were performed by using PBS with 0.1% Tween 20 (pH 7.4). The serum was diluted in PBS and analyzed in duplicate, and then biotin-conjugated goat anti-mouse heavy- and light-chain antibodies were added, followed by europium-labeled streptavidin (Delfia, Wallac OY, Turku, Finland), and enhancement solution (Delfia Wallac); the amount of antibody was detected by dissociation-enhanced time-resolved fluoroimmunoassays research fluorometer. Serum samples from COMP-induced arthritis mice were used as a positive control. Antibody titers against CII in serum were determined by sandwich ELISA similar to COMP antibody assay, except the plates were coated with 10 μg/mL CII [28]. Antibody levels are shown as fluorescence units.

### Determination of serum levels of cartilage oligomeric matrix protein

Serum concentration of COMP was determined by a competitive ELISA [3]. Rat COMP was used for coating the microtiter plates and for preparing the standard curve included in each plate. Plates were blocked with 1% BSA in PBS for 2 hours at room temperature. After blocking, serum samples were co-incubated with rabbit polyclonal antiserum against rat COMP (generously provided by Dick Heinegård, Section for Connective Tissue Biology, Lund University) and incubated for 2 hours at room temperature. The amount of COMP was estimated after incubation with an alkaline phosphatase-conjugated swine anti-rabbit isotype-specific antibody (DakoCytomation, Glostrup, Denmark) and phosphatase substrate (Sigma-Aldrich, St. Louis, MO, USA) as substrate followed by detection at 405 nm in a Spectra Max plate reader (Molecular Devices Corporation, Sunnyvale, CA, USA).

### Statistics

Quantitative data are expressed as mean ± standard error of the mean, and significance analysis of disease onset was performed by using the Student *t *test. Severity comparison was performed by the Mann-Whitney *U *test. All results obtained from COMP-deficient mice were compared with those obtained from B10.Q wild-type littermate controls. Differences were considered to be statistically significant for *P *values of less than 0.05.

## Results

### Generation of B10.Q cartilage oligomeric matrix protein-deficient mice

To determine a possible effect of COMP deficiency on CIA and CAIA, we backcrossed COMP-deficient 129/Sv mice with B10.Q mice. The B10.Q mouse has a C57BL/10 genetic background and a DBA/1-derived congenic fragment containing the MHC class II gene A^q ^molecule allowing an immune response to CII [29]. The experiments were performed in animals after backcrossing for 10 generations to B10.Q mice. Remaining differences in the genome background were excluded by littermate-controlled experiments. COMP-deficient mice in B10.Q background have no microscopic or macroscopic sign of osteoarthritis or other pathologies in a large number of normal young and old (more than 1 year) mice (data not shown).

### Early onset and increased severity in chronic phase of collagen II-induced arthritis in cartilage oligomeric matrix protein-deficient mice

**Figure 1:**
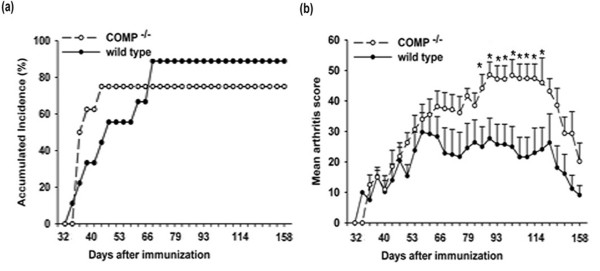
Cartilage oligomeric matrix protein (COMP)-deficient B10.Q mice show earlier onset and more severe collagen II (CII)-induced arthritis. Arthritis was induced on day 0 by a subcutaneous injection of rat CII emulsified in complete Freund's adjuvant in COMP-deficient mice and B10.Q mice. The mice were boosted on day 30 with an injection of rat CII in incomplete Freund's adjuvant. Arthritis severity was followed for 158 days. Arthritis incidence **(a) **and mean arthritis score **(b) **are indicated. The data are representative of three experiments. Asterisks indicate significant differences between COMP-deficient mice (n = 8) and wild-type mice (n = 9) (*P *< 0.05).

To test whether COMP deficiency makes the cartilage more susceptible to CIA, we immunized the mice with heterologous rat CII. The male mice in the COMP-deficient and wild-type groups started to develop arthritis on day 32, and the disease course could be divided into two phases, acute phase (from days 32 to 66) and chronic phase (from days 66 to 158), because there was a decrease in the mean arthritis score after day 66 in wild-type mice. COMP deficiency led to early onset of the disease, with a mean onset of arthritis at 37.5 ± 2.81 days in COMP-deficient mice compared with 48.4 ± 13.7 days in the wild-type littermate group (*P *< 0.05). There was no change in severity during the acute phase between COMP-deficient mice and wild-type mice (Figure 1). In the chronic phase of the disease course, the mean arthritis score in COMP-deficient mice continued to increase. COMP-deficient mice developed significantly more severe arthritis during the chronic phase of the disease course compared with wild-type mice (Figure 1). The incidence of arthritis in COMP-deficient male mice (75%) was not significantly different compared with wild-type mice (88.8%). For the female mice, both COMP-deficient and wild-type mice were less susceptible to CIA: only two of eight COMP-deficient female mice developed arthritis, and two of nine wild-type mice developed arthritis. Because of this low incidence of arthritis, it was not possible to compare the onset day and the severity of arthritis developed among the female mice.

### Cartilage oligomeric matrix protein deficiency did not alter collagen II-specific antibody synthesis

**Figure 2:**
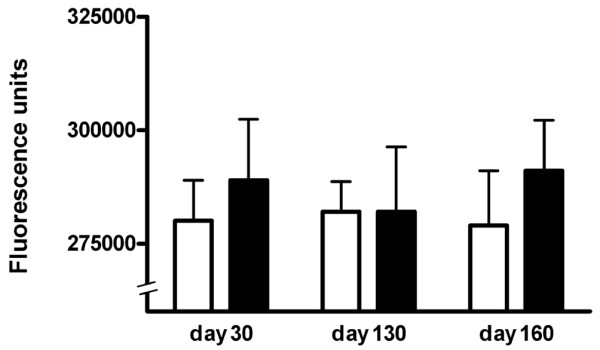
Collagen II (CII) antibody levels in cartilage oligomeric matrix protein (COMP)-deficient and wild-type mice. Serum was taken at days 0, 30, 130, and 160 after CII immunization and analyzed for CII antibody levels. No CII antibodies were detected in wild-type or COMP-deficient mice at day 0. Data are representative of three experiments on COMP-deficient mice (n = 8) and wild-type mice (n = 9). Filled bars (COMP-deficient) and open bars (wild-type) show mean values and standard error (error bars).

Antibodies have been shown to play an important role in arthritis onset and the severity of the disease [30,31]. Both COMP-deficient mice and wild-type mice mounted high-antibody titers to CII. Antibody levels, however, were found to be similar in the COMP-deficient and wild-type mice at days 30, 130, and 160 (Figure 2), demonstrating that COMP deficiency in cartilage had no significant effect on CII-specific antibody response.

### Antibodies to cartilage oligomeric matrix protein did not play a role in disease induction

**Figure 3:**
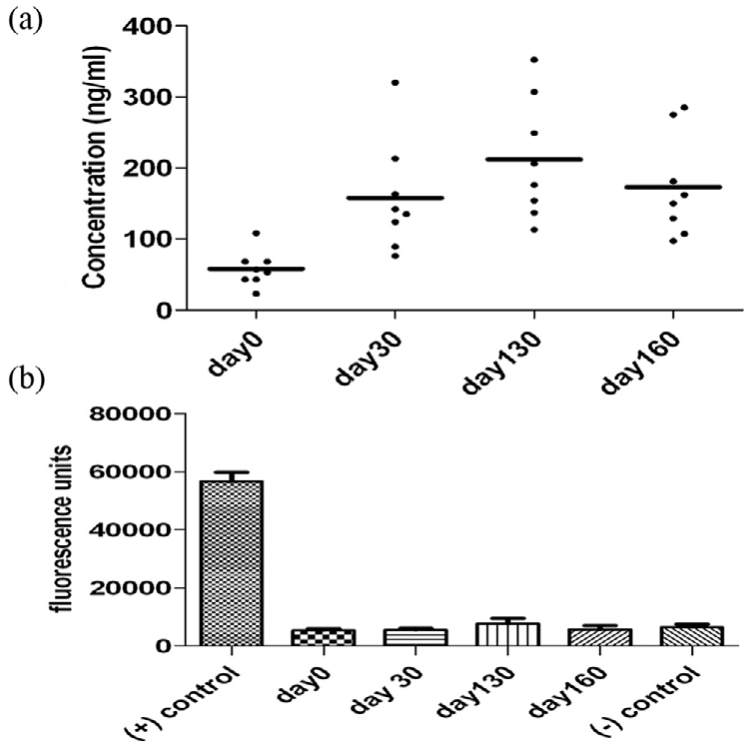
Serum cartilage oligomeric matrix protein (COMP) and COMP antibody levels in mice with collagen II-induced arthritis (CIA) (n = 8). Serum from wild-type B10.Q mice with CIA was taken at days 0, 30, 130, and 160 after collagen II immunization. COMP **(a) **and COMP antibodies **(b) **in serum were analyzed. The negative (-) control was serum from COMP-deficient mice (n = 7), and the positive (+) control was serum from COMP-deficient mice injected with COMP (n = 7).

The serum COMP level is used as a biomarker both in humans and in experimental animals to detect ongoing inflammation in the joints as well as a measure of severity of the arthritis induced [3-5]. Hence, we measured the COMP levels in arthritic animals and found released COMP fragments in the serum collected from arthritic wild-type mice (Figure 3a). However, we could not detect antibodies to COMP in the serum at any point during the arthritic disease course from days 30 to 160, suggesting that antibodies to COMP are not involved in the pathological process of CIA (Figure 3b).

### Cartilage oligomeric matrix protein-deficient mice and wild-type mice showed similar responses to collagen II antibody-induced arthritis

**Figure 4:**
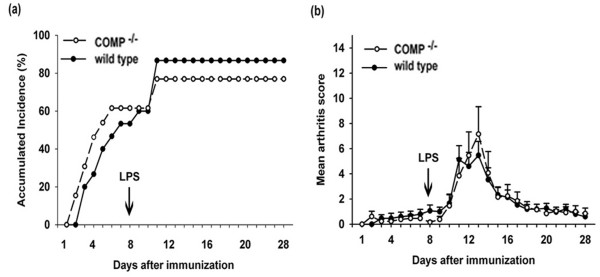
Collagen antibody-induced arthritis (CAIA) in cartilage oligomeric matrix protein (COMP)-deficient and wild-type B10.Q mice. CAIA was induced by intravenous transfer of cocktail of four monoclonal antibodies reacting with collagen II epitopes (CIIC1, M2139, CIIC2, and UL1) on day 0, and lipopolysaccharide (LPS) (25 μg per mouse) was injected intraperitoneally on day 8. Mice were monitored for arthritis development on the indicated days. Arthritis incidence **(a) **and mean arthritis score **(b) **are indicated. Data were obtained from two identically performed experiments: COMP-deficient mice (n = 13) and wild-type mice (n = 15).

To induce CAIA, the mice were injected with a standard cocktail of CIIC1, M2139, CIIC2, and UL1 monoclonal antibodies directed against dominant B-cell epitopes of CII. We observed a possible influence on disease onset in COMP-deficient mice, with the mean onset day in COMP-deficient mice of 5.1 ± 3.35 days compared with 6.85 ± 3.4 days in control mice. This difference was not significant. Consistent with CIA results, no difference was found in the incidence of arthritis between COMP-deficient and wild-type littermate controls (Figure 4a). Both groups developed arthritis with a peak around day 12, which subsided with the same rate. There was no difference in mean arthritis score between these two groups during the rapid phase of disease progress (Figure 4b).

## Discussion

COMP is a major non-collagenous component of cartilage and contributes about 1% of the wet weight of articular cartilage [15]. COMP has been studied intensively due to the fact that COMP mutations are associated with musculoskeletal disease [18,19]. However, the biological function of COMP in cartilage remains unknown. We have previously generated COMP-null mice and shown that they have normal skeletal development. This raises the argument that COMP in cartilage may be functionally redundant

Here, we show that COMP-deficient mice develop an early-onset CIA and more severe arthritis during the chronic phase of the disease.

The findings that COMP-deficient mice develop severe autoimmune CIA indicate that COMP deficiency makes the cartilage more susceptible to an inflammatory attack. Antibodies play a critical role in the initiation of CIA [30,31]. The COMP-deficient mice developed the same high levels of anti-CII antibodies as wild-type mice during CIA. We have previously reported that collagen IX-deficient mice are more susceptible to CAIA, most likely due to the higher penetrance of anti-CII antibodies into cartilage [24]. Accordingly, there might be a difference in accessibility of antibodies to cartilage matrix due to COMP deficiency. However, the susceptibility to CAIA was not enhanced in COMP-deficient mice although there is a possible trend toward an earlier onset. COMP, however, is released systemically during CIA and it is likely that these fragments affect T cell-dependent immune regulation, a phenomenon that may be different in COMP-deficient mice. The nature of the T-cell response in wild-type versus COMP-deficient animals requires another set of experiments as the circulating COMP affects T-cell tolerance, and we therefore need to determine the major T-cell epitopes. Again, since there were no changes in anti-CII antibody titers and anti-CII antibodies are T cell-dependent, the T-cell responsiveness as such is not an obvious explanation.

**Figure 5:**
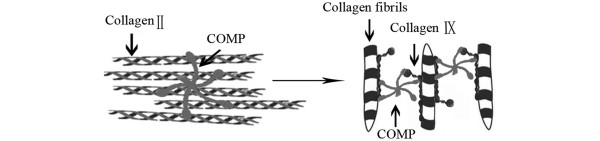
Schematic picture of interactions of cartilage oligomeric matrix protein (COMP) with collagen II and collagen IX. In cartilage, collagen II is the main collagen and makes up a fiber network that provides a cohesive framework. COMP binds via each C-terminal globule to triple-helical collagen II with high affinity. The binding between COMP and triple-helical collagen domains influences collagen fibril assembly. In addition, interactions between COMP and collagen IX provide the potential for crossbridging collagen fibrils to form a cartilage collagen network. Figure is modified from Heinegård and colleagues [35].

It has been reported that pentameric COMP binds to collagen I/collagen II [32] and collagen IX [33] with high affinity via the C-terminal globular domains (Figure 5). Indeed, COMP appears to function as an accelerator of collagen fibril formation [34]. These COMP-collagen interactions may be crucial for the formation of a cartilage collagen network. It is possible that COMP deficiency leads to instability and changed exposure of concealed epitopes, and it would be interesting to study whether new epitopes on CII and CIX indeed are exposed and, if so, whether this could mediate pathologic changes in COMP-deficient mice. Furthermore, COMP may have direct effects on chondrocytes (for example, through interaction with integrins [21]), which may regulate chondrocyte cellular activities and phenotypic development. These functions of COMP might be especially important during remodeling of cartilage after injury or during inflammatory conditions. In the present study, the observation that COMP-deficient mice show a more severe arthritis during the chronic phase of CIA, but not during the acute phase, supports the hypothesis that COMP is important in cartilage repair processes and thus in cartilage regeneration and remodeling.

The primary target cartilage antigen in CIA is CII, which initiates the autoimmune reaction leading to arthritis. In the course of the disease, when the erosion of the cartilage is taking place, it is possible that immune reactions to other cartilage proteins are initiated and contribute to the disease course. In the CIA model, COMP was found to be released to serum. Using COMP-deficient mice as a negative control and mice immunized with COMP to induce arthritis as a positive control, we investigated whether there was an immune response against COMP during CIA. We did not find COMP antibodies at any point during the whole disease course, suggesting no involvement of immune response against COMP in CII-induced arthritis.

## Conclusion

COMP deficiency in mice subjected to CIA did not affect either incidence or anti-CII antibody titers but caused a significant early onset and increase in the severity of the disease during the chronic phase of arthritis. Arthritic B10.Q mice suffering from CIA did not respond immunologically to COMP by means of COMP antibody synthesis. Results of the CAIA study demonstrate that antibodies accessed CII epitopes similar in COMP-deficient and COMP-sufficient mice. Our results emphasize the importance of COMP in cartilage stability, and the mechanism underlying the exacerbation of CIA in COMP-deficient mice is assumed to be found in COMP-dependent changes in the cartilage erosion/repair process.

## Abbreviations

CAIA: collagen II antibody-induced arthritis; CIA: collagen-induced arthritis; CII: collagen II; COMP: cartilage oligomeric matrix protein; ELISA: enzyme-linked immunosorbent assay; LPS: lipopolysaccharide; MED: multiple epiphyseal dysplasia; MHC: major histocompatibility complex; PBS: phosphate-buffered saline; PCR: polymerase chain reaction; PSACH: pseudoachondroplasia; RA: rheumatoid arthritis; TSP: thrombospondin.

## Competing interests

The authors declare that they have no competing interests.

## Authors' contributions

HG was responsible for the majority of the practical work and the writing of the manuscript. The study was originally designed by RM, AA, ÅO, and SC in collaboration with RH. KSN helped with CAIA experiments. All authors were involved in different methodological parts, the interpretation of data, and the writing of the manuscript. All authors read and approved the final manuscript.

## References

[B1] Feldmann M, Brennan FM, Maini RN (1996). Rheumatoid arthritis. Cell.

[B2] Arnett FC, Edworthy SM, Bloch DA, McShane DJ, Fries JF, Cooper NS, Healey LA, Kaplan SR, Liang MH, Luthra HS, Medsger TA, Mitchell DM, Neustadt DH, Pinals RS, Schaller JG, Sharp JT, Wilder RL, Hunder GG (1988). The American Rheumatism Association 1987 revised criteria for the classification of rheumatoid arthritis. Arthritis Rheum.

[B3] Saxne T, Heinegård D (1992). Cartilage oligomeric matrix protein: a novel marker of cartilage turnover detectable in synovial fluid and blood. Br J Rheumatol.

[B4] Neidhart M, Hauser N, Paulsson M, DiCesare PE, Michel BA, Hauselmann HJ (1997). Small fragments of cartilage oligomeric matrix protein in synovial fluid and serum as markers for cartilage degradation. Br J Rheumatol.

[B5] Mansson B, Carey D, Alini M, Ionescu M, Rosenberg LC, Poole AR, Heinegård D, Saxne T (1995). Cartilage and bone metabolism in rheumatoid arthritis. Differences between rapid and slow progression of disease identified by serum markers of cartilage metabolism. J Clin Invest.

[B6] Trentham DE, Townes AS, Kang AH (1977). Autoimmunity to type II collagen an experimental model of arthritis. J Exp Med.

[B7] Carlsen S, Hansson AS, Olsson H, Heinegård D, Holmdahl R (1998). Cartilage oligomeric matrix protein (COMP)-induced arthritis in rats. Clin Exp Immunol.

[B8] Oldberg A, Antonsson P, Lindblom K, Heinegård D (1992). COMP (cartilage oligomeric matrix protein) is structurally related to the thrombospondins. J Biol Chem.

[B9] Coligan JE, Slayter HS (1984). Structure of thrombospondin. J Biol Chem.

[B10] Lawler J, Derick LH, Connolly JE, Chen JH, Chao FC (1985). The structure of human platelet thrombospondin. J Biol Chem.

[B11] Laherty CD, O'Rourke K, Wolf FW, Katz R, Seldin MF, Dixit VM (1992). Characterization of mouse thrombospondin 2 sequence and expression during cell growth and development. J Biol Chem.

[B12] Bornstein P, O'Rourke K, Wikstrom K, Wolf FW, Katz R, Li P, Dixit VM (1991). A second, expressed thrombospondin gene (Thbs2) exists in the mouse genome. J Biol Chem.

[B13] Vos HL, Devarayalu S, de Vries Y, Bornstein P (1992). Thrombospondin 3 (Thbs3), a new member of the thrombospondin gene family. J Biol Chem.

[B14] Lawler J, Duquette M, Whittaker CA, Adams JC, McHenry K, DeSimone DW (1993). Identification and characterization of thrombospondin-4, a new member of the thrombospondin gene family. J Cell Biol.

[B15] Hedbom E, Antonsson P, Hjerpe A, Aeschlimann D, Paulsson M, Rosa-Pimentel E, Sommarin Y, Wendel M, Oldberg A, Heinegård D (1992). Cartilage matrix proteins. An acidic oligomeric protein (COMP) detected only in cartilage. J Biol Chem.

[B16] DiCesare P, Hauser N, Lehman D, Pasumarti S, Paulsson M (1994). Cartilage oligomeric matrix protein (COMP) is an abundant component of tendon. FEBS Lett.

[B17] Shen Z, Heinegård D, Sommarin Y (1995). Distribution and expression of cartilage oligomeric matrix protein and bone sialoprotein show marked changes during rat femoral head development. Matrix Biol.

[B18] Briggs MD, Hoffman SM, King LM, Olsen AS, Mohrenweiser H, Leroy JG, Mortier GR, Rimoin DL, Lachman RS, Gaines ES, Cekleniak JA, Knowlton RG, Cohn DH (1995). Pseudoachondroplasia and multiple epiphyseal dysplasia due to mutations in the cartilage oligomeric matrix protein gene. Nat Genet.

[B19] Hecht JT, Nelson LD, Crowder E, Wang Y, Elder FF, Harrison WR, Francomano CA, Prange CK, Lennon GG, Deere M, Lawler J (1995). Mutations in exon 17B of cartilage oligomeric matrix protein (COMP) cause pseudoachondroplasia. Nat Genet.

[B20] McKeand J, Rotta J, Hecht JT (1996). Natural history study of pseudoachondroplasia. Am J Med Genet.

[B21] Chen FH, Thomas AO, Hecht JT, Goldring MB, Lawler J (2005). Cartilage oligomeric matrix protein/thrombospondin 5 supports chondrocyte attachment through interaction with integrins. J Biol Chem.

[B22] Beighton P, Giedion ZA, Gorlin R, Hall J, Horton B, Kozlowski K, Lachman R, Langer LO, Maroteaux P, Poznanski A, Rimoin DL, Sillence D, Spranger J (1992). International classification of osteochondrodysplasias. International Working Group on Constitutional Diseases of Bone. Am J Med Genet.

[B23] Svensson L, Aszodi A, Heinegård D, Hunziker EB, Reinholt FP, Fassler R, Oldberg A (2002). Cartilage oligomeric matrix protein-deficient mice have normal skeletal development. Mol Cell Biol.

[B24] Carlsen S, Nandakumar KS, Holmdahl R (2006). Type IX collagen deficiency enhances the binding of cartilage-specific antibodies and arthritis severity. Arthritis Res Ther.

[B25] Smith BD, Martin GR, Miller EJ, Dorfman A, Swarm R (1975). Nature of the collagen synthesized by a transplanted chondrosarcoma. Arch Biochem Biophys.

[B26] Holmdahl R, Carlsen S, Mikulowska A, Vestberg M, Brunsberg U, Hansson A-S, Sundvall M, Jansson L, Pettersson U, Adolph KW (1998). Genetic analysis of mouse models for rheumatoid arthritis. Human Genome Methods.

[B27] Nandakumar KS, Holmdahl R (2005). Efficient promotion of collagen antibody induced arthritis (CAIA) using four monoclonal antibodies specific for the major epitopes recognized in both collagen induced arthritis and rheumatoid arthritis. J Immunol Methods.

[B28] Liljander M, Sallstrom MA, Andersson S, Andersson A, Holmdahl R, Mattsson R (2006). Identification of collagen-induced arthritis loci in aged multiparous female mice. Arthritis Res Ther.

[B29] Holmdahl R, Karlsson M, Andersson ME, Rask L, Andersson L (1989). Localization of a critical restriction site on the I-A beta chain that determines susceptibility to collagen-induced arthritis in mice. Proc Natl Acad Sci USA.

[B30] Bajtner E, Nandakumar KS, Engstrom A, Holmdahl R (2005). Chronic development of collagen-induced arthritis is associated with arthritogenic antibodies against specific epitopes on type II collagen. Arthritis Res Ther.

[B31] Nandakumar KS, Johansson BP, Bjorck L, Holmdahl R (2007). Blocking of experimental arthritis by cleavage of IgG antibodies *in vivo*. Arthritis Rheum.

[B32] Rosenberg K, Olsson H, Morgelin M, Heinegård D (1998). Cartilage oligomeric matrix protein shows high affinity zinc-dependent interaction with triple helical collagen. J Biol Chem.

[B33] Holden P, Meadows RS, Chapman KL, Grant ME, Kadler KE, Briggs MD (2001). Cartilage oligomeric matrix protein interacts with type IX collagen, and disruptions to these interactions identify a pathogenetic mechanism in a bone dysplasia family. J Biol Chem.

[B34] Halasz K, Kassner A, Morgelin M, Heinegård D (2007). COMP acts as a catalyst in collagen fibrillogenesis. J Biol Chem.

[B35] Heinegård D, Lorenzo P, Saxne T, Hochberg MC, Silman AJ, Smolen JS, Weinblatt ME, Weisman MH (2008). Cell Biology, Biochemistry, and Molecular Biology of Articular Cartilage. Rheumatology.

